# Long term clinical impact of successful recanalization of chronic total occlusion in patients with and without type 2 diabetes mellitus

**DOI:** 10.1186/s12933-020-01093-6

**Published:** 2020-08-01

**Authors:** Chuan-Tsai Tsai, Wei-Chieh Huang, Hsin-I Teng, Yi-Lin Tsai, Tse-Min Lu

**Affiliations:** 1 Division of Cardiology, Department of Medicine, Taipei Veterans General Hospital, No. 201, Sec. 2, Shih-Pai Road, Taipei, Taiwan; 2 Cardiovascular Research Center, National Yang-Ming University, Taipei, Taiwan; 3 Institute of Clinical Medicine, National Yang-Ming University, Taipei, Taiwan

**Keywords:** Chronic total occlusion, Percutaneous coronary intervention, Diabetes mellitus

## Abstract

**Background:**

Diabetes mellitus is one of the risk factors for coronary artery disease and frequently associated with multivessels disease and poor clinical outcomes. Long term outcome of successful revascularization of chronic total occlusions (CTO) in diabetes patients remains controversial.

**Methods and results:**

From January 2005 to December 2015, 739 patients who underwent revascularization for CTO in Taipei Veterans General Hospital were included in this study, of which 313 (42%) patients were diabetes patients. Overall successful rate of revascularization was 619 (84%) patients whereas that in diabetics and non-diabetics were 265 (84%) and 354 (83%) respectively. Median follow up was 1095 days (median: 5 years, interquartile range: 1–10 years). During 3 years follow-up period, 59 (10%) in successful group and 18 (15%) patients in failure group died. Although successful revascularization of CTO was non-significantly associated with better outcome in total cohort (hazard ratio (HR): 0.593, 95% confidence interval (CI) 0.349–0.008, P: 0.054), it might be associated with lower risk of all-cause mortality (HR: 0.307, 95% CI 0.156–0.604, P: 0.001) and CV mortality (HR: 0.266, 95% CI 0.095–0.748, P: 0.012) in diabetics (P: 0.512). In contrast, successful CTO revascularization didn’t improve outcomes in non-diabetics (all P > 0.05). In multivariate cox regression analysis, successful CTO revascularization remained an independent predictor for 3-years survival in diabetic subgroup (HR: 0.289, 95% CI 0.125–0.667, P: 0.004). The multivariate analysis result was similar after propensity score matching (all-cause mortality, HR: 0.348, 95% CI 0.142–0.851, P: 0.021).

**Conclusions:**

Successful CTO revascularization in diabetes may be related to better long term survival benefit but not in non-diabetic population.

## Background

Approximately 15% to 30% of patients who received coronary angiography had one or multiple chronic total occlusion (CTO) of coronary arteries [1, 2]. Percutaneous coronary intervention (PCI) for CTO is technical challenging and always need ample experiences, dedicated techniques and advanced interventional devices. Moreover, PCI for CTO lesion is related to higher radiation exposure to the patient and operator, more contrast volumes and increased risk for peri-procedural complications. Successful PCI of CTO lesion has been reported to be associated with improved left ventricular function and better clinical outcomes; while other studies showed negative results [3, 4]. On the other hand, Diabetes mellitus, a well-established risk factor of atherosclerosis, is always associated with more complex atherosclerotic coronary artery disease, including multi-vessel disease, diffuse and small vessel disease, and heavily calcified lesions. In addition, diabetes has been reported to be associated with longer length of CTO lesions [5, 6], and the treatment for CTO lesions in diabetes are more complex with lower success rate [7]. Even after successful revascularization, there were higher in-hospital major adverse cardiovascular and cerebrovascular events (MACCE) in diabetic patients comparing to non-diabetic patients [8]. Besides, diabetes is related to higher incidence of mortality and revascularization after CTO PCI up to 5 years compared to non-diabetics [9]. However, long term impact of successful revascularization for CTO lesions in diabetes population remains unknown and controversial. Therefore, in this study we aimed to investigate the long-term clinical outcomes in diabetic patients undergoing CTO PCI.

## Methods

### Study population

We enrolled patients who underwent CTO PCI at Taipei Veterans General Hospital. From January 2005 to December 2015, a total of 739 patients underwent intervention for CTO lesions. CTO lesions were defined as complete blockage of a coronary artery for longer than 3 months with thrombolysis in myocardial infarction (TIMI) 0. All patients had at least 1 CTO lesion and met the indication of recanalization of CTO lesion. Indications for CTO revascularization were as follows: (1) angina resistant to pharmacological therapy, (2) exercise-induced symptoms or (3) exercise induced evidence of myocardial ischemia. Patients with acute coronary syndrome and end stage renal disease on renal replacement therapy were excluded. Diabetes mellitus (DM) was defined as a fasting plasma glucose of at least 126 mg/dl, or 2 h postprandial plasma glucose of 200 mg/dl or glycated hemoglobin of at least 6.5% or random plasma glucose of at least 200 mg/dl in presence of classic symptoms of hyperglycemia [10]. Left ventricular ejection fraction was measured from transthoracic echocardiography or left ventriculography. Renal function was classified according to estimated glomerular filtration rate (eGFR) calculated by the modified diet in renal disease equation for Chinese (MDRDc) [11]. Patient’s demographics, coronary angiography, PCI records, in-hospital treatments, and in-hospital laboratory tests were extracted from web based electronic medical system of our hospital.

### Coronary angiography and percutaneous coronary intervention (PCI) procedure

Diagnostic coronary angiography was evaluated carefully by experience cardiologists for the morphology of CTO lesion and collaterals. J-CTO score was calculated as previously reported [12]. Radial or femoral artery approaches/uni- or bi-directional approaches were used for diagnostic angiography and percutaneous coronary intervention according to standardized protocol of cardiac catheterization laboratory. After unfractionated heparin (10,000 IU bolus) was administered before the procedure to achieve an activated clotting time > 300 s, we routinely tried antegrade approach first. Antegrade approach includes single wire technique with wire escalation and parallel wire technique. If antegrade approach did not work, we would try retrograde approach if there are suitable collaterals available. After wire crossing collateral retrogradely, we always tried retrograde wiring technique, kissing wire technique, and reverse controlled antegrade and retrograde subintimal tracking (CART) technique. However, if both approaches failed and the CTO lesion morphology is suitable, intravascular ultrasound (IVUS) guided wiring re-entry technique would be tried. The PCI procedure was considered angiographically successful if residual stenosis < 30% and coronary Thrombolysis in Myocardial Infarction grade 3 flow were obtained at the end of the procedure.

Dual antiplatelets therapy was started on the day before PCI procedure or immediately after the procedure, and all patients received aspirin (100 mg/day) indefinitely and clopidogrel (300 to 600 mg loading dose, and 75 mg maintenance per day) for at least 3 month (bare metal stent (BMS)) or 12 months (drug-eluting stent, DES). After 1 year, aspirin or clopidogrel was maintained life-long. Medications for treatment of angina pectoris (calcium channel blockers, beta-blockers and nitrates) were continued.

### Clinical outcomes

Clinical endpoints were 3 years all-cause mortality, cardiovascular (CV) mortality, nonfatal myocardial infarct (MI) and composite endpoints (MACE). MACE was the composite endpoint of all-cause mortality, CV mortality and nonfatal MI. Myocardial infarction was defined as the presence of significant new Q waves in at least 2 electrocardiographic leads or of symptoms compatible with MI associated with increase in creatine kinase-MB fraction ≥ 3 times the upper limit of the reference range. Periprocedural cardiac enzymes elevation was excluded from this definition of MI. Cardiovascular death was diagnosed as any death with definite cardiovascular cause or any death that was not clearly attributed to a non-cardiovascular cause. Hospital re-admission and outpatient clinic records from our hospital web-based system were obtained for clinical outcomes. In addition, patients were contacted by research coordinator by telephone interview at the end of study period if loss of follow up in our hospital. The study protocol was approved by the Institutional Review Board at Taipei-Veterans General Hospital, and all participants provided written informed consent.

### Statistical analysis

Continuous variables were compared with Student’s t-test and were expressed as mean ± standard deviation (SD). Categorical data were tested using Chi-square test and presented as frequencies and percentages. Propensity score matching was performed using logistic regression model. We adjusted variables that were known as confounding factors (age, sex, renal function). Success and failure to revascularize groups were matched by a 4:1 matching protocol according to propensity scores with the width equal to 0.05 of the standard deviation. Kaplan–Meier estimates were used for survival curves which were compared with log-rank test. Multivariate analyses were performed with a cox proportional hazards model and confounders were selected according to statistically significance (P < 0.05) in univariate analysis which were age, renal function, prior stroke, peripheral arterial disease, renal function and left ventricular ejection fraction (LVEF). P-value of less than 0.05 was considered as statistically significance. All statistically analyses were performed with the use of SPSS 17.0 software (SPSS Inc, Chicago, IL, USA).

## Results

**Fig. 1:**
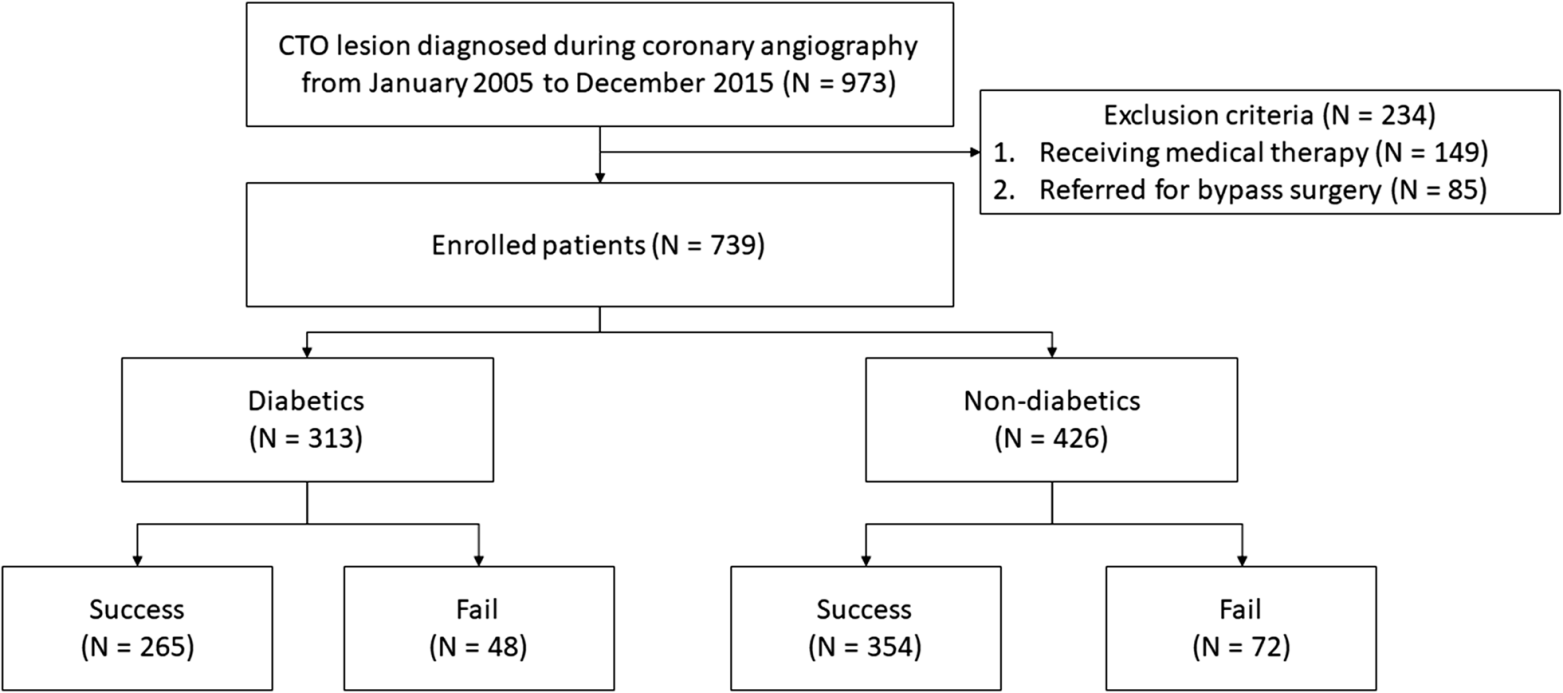
Flowchart for patients enrollment

From January 2005 to December 2015, a total of 973 patients were found to have CTO lesion during coronary angiography in which 739 patients received revascularization at Taipei Veterans General Hospital (Fig. 1). The mean age was 68 ± 13 years, and most of the patients were male (675, 91%). Most patients had multi-vessel disease (607, 82%), and right coronary artery was the most common treated CTO vessel (362, 49%). 76 (10%) patients underwent bypass surgery prior to CTO PCI. The mean J-CTO score was 2.5 ± 0.97. Chronic kidney disease was present in 165 patients (22%). Most CTO lesions were crossed by antegrade wire escalation technique/parallel wire technique, while the retrograde approach was tried in only 56 patients (7.6%). Successful revascularization was achieved in 619 (84%) patients of entire cohort. Compared to patients with successful revascularization, the failed PCI group had poorer renal function, higher prevalence of multivessel CTO, and longer CTO length. Drug eluting stent was used in 317 (43%) patients. The baseline clinical and angiographic characteristics are shown in Table 1.

**Table 1:** Baseline demographics and angiographic characteristics of entire population with successful and Failed revascularization

	Entire population (n = 739)		
	Success (n = 619)	Failed (n = 120)	P value
Age (years)	67 ± 13	69 ± 13	0.14
Gender (male)	568 (92)	107 (89)	0.38
Hypertension	467 (75)	96 (80)	0.35
DM	265 (43)	48 (40)	0.61
Hyperlipidemia	267 (43)	53 (44)	0.84
Prior stroke	51 (8)	11 (9)	0.72
PAD	34 (6)	11 (9)	0.14
Smoking	253 (41)	47 (39)	0.76
Prior MI	135 (22)	24 (20)	0.72
Prior PCI	317 (51)	66 (55)	0.49
Prior CABG	61 (10)	15 (13)	0.41
LVEF (%)	49 ± 12	48 ± 13	0.61
eGFR, MDRDc (ml/min)	84 ± 33	76 ± 34	0.01
LDL (mg/dl)	102 ± 35	97 ± 32	0.19
HbA1C (%)	8.0 ± 3.8	7.6 ± 1.7	0.51
Location of CTO			
LAD	271 (44)	46 (38)	0.31
LCx	159 (26)	37 (31)	0.26
RCA	293 (47)	63 (53)	0.32
J CTO score	2.45 ± 1.00	2.55 ± 0.96	0.32
MVD	501 (81)	106 (88)	0.07
Lesion length (mm)	38 ± 18	32 ± 11	0.01
Lesion width (mm)	3.2 ± 3.6	3.0 ± 0.5	0.73
Primary retrograde	48 (8)	8 (7)	0.34

Values are given as mean and standard deviation or numbers and percentages

*DM* diabetes mellitus, *PCI* percutaneous coronary intervention, *PAD* peripheral arterial disease, *MI* myocardial infarct, *PCI* percutaneous coronary intervention, *CABG* coronary artery bypass graft surgery, *LVEF* left ventricular ejection fraction, *eGFR* estimated glomerular filtration rate, *MDRDc* modification of diet in renal disease Chinese, *LDL* low density lipoprotein, *CTO* chronic total occlusion, *CAD* coronary artery disease, *LAD* left anterior descending, *LCx* left circumflex, *RCA* right coronary artery, *MVD* multivessel significant coronary artery disease

**Fig. 2:**
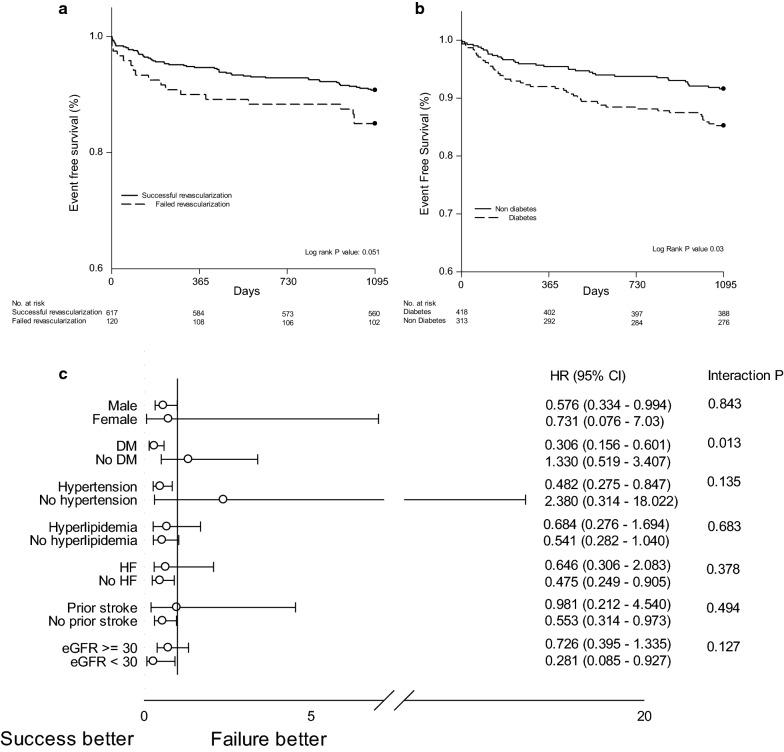
Kaplan Meier survival curves for 3-years all cause mortality of **a** entire population, **b** Diabetes vs non diabetes, **c** subgroup analysis of entire population

The incidences of clinical outcomes (all-cause mortality, CV mortality, nonfatal MI and MACE) followed up for 3 years (median: 5 years, interquartile range: 1–10 years) were summarized in Table 2 (Fig. 2a). In entire population, there were no significant differences in the incidence of all-cause mortality, CV mortality, nonfatal MI and MACE (hazard ratio (HR): 0.593, 95% confidence interval (CI) 0.349–1.008, P: 0.054; HR: 0.472, 95% CI 0.217–1.024, P: 0.057; HR: 0.867, 95% CI 0.294–2.563, P: 0.797; HR: 0.734, 95% CI 0.449–1.200, P: 0.218 respectively) between successful revascularization group and failed revascularization group. Three years all-cause mortality was statistically significant higher in diabetes population compared to non-diabetes (P: 0.03) (Fig. 2b). Subgroup analysis showed that successful revascularization in diabetic population exhibited better survival benefit compared to that of non-diabetics (HR: 0.306, 95% CI 0.156–0.601 vs HR: 1.330, 95% CI 0.519–3.407, interaction P: 0.013) (Fig. 2c).

**Table 2:** Various clinical outcomes up to 3 years by Kaplan–Meier curved analysis

Entire population	Incidence of event at 3 years [n (%)]			
	Procedure		HR (95% CI)	P value
	Successful PCI (n = 619)	Failed PCI (n = 120)		
All cause mortality	59 (10)	18 (15)	0.593 (0.349–1.008)	0.054
CV mortality	22 (4)	9 (8)	0.472 (0.217–1.024)	0.057
Nonfatal MI	18 (3)	4 (3)	0.867 (0.294–2.563)	0.797
MACE	80 (13)	20 (17)	0.734 (0.449–1.200)	0.218

Diabetes patients	Successful PCI (n = 265)	Failed PCI (n = 48)		
All cause mortality	25 (9)	13 (27)	0.307 (0.156–0.604)	0.001
CV mortality	9 (3)	6 (13)	0.266 (0.095–0.748)	0.012
Nonfatal MI	11 (4)	3 (6)	0.652 (0.182–2.338)	0.512
MACE	39 (15)	14 (29)	0.454 (0.246–0.837)	0.011

Non diabetes patients	Successful PCI (n = 354)	Failed PCI (n = 72)		
All cause mortality	34 (10)	4 (7)	1.334 (0.521–3.417)	0.548
CV mortality	13 (4)	3 (4)	0.885 (0.252–3.107)	0.849
Nonfatal MI	7 (2)	1 (1)	1.423 (0.175–11.565)	0.741
MACE	41 (12)	6 (8)	1.351 (0.573–3.188)	0.491

Diabetes patients after matching	Successful PCI (n = 188)	Failed PCI (n = 47)		
All cause mortality	21 (11)	12 (26)	0.386 (0.188–0.789)	0.009
CV mortality	7 (4)	6 (13)	0.268 (0.090–0.798)	0.018
Nonfatal MI	8 (4)	3 (6)	0.584 (0.154–2.210)	0.429
MACE	28 (15)	13 (28)	1.511 (0.338–6.753)	0.589

*CV* cardiovascular, *MI* myocardial infarct, *MACE* major adverse cardiovascular events (defined as the composite of all-cause mortality, cardiovascular mortality, and non fatal myocardial infarct)

Totally 313 (42%) patients are diabetics and 426 (58%) patients are non-diabetics. Among diabetic patients, 68 (21.7%) patients received insulin treatment. Compared to non-diabetic patients, diabetic patients were significantly older, with higher percentage of hypertension, worse renal function, multi-vessels disease, and reduced LVEF. There was no statistically difference in J-CTO score between two groups of diabetics and non-diabetics. Syntax score was statistically significant higher in failed group of diabetics but not different in non-diabetics. However, the revascularization successful rate was similar between diabetic patients (265, 84%), and non-diabetics patients (354, 83%, P = 0.614). The angiographic procedure and characteristics were not significantly different between the two groups (Table 3).

**Table 3:** Baseline demographics and angiographic characteristics of diabetes and non diabetes population with successful and failed revascularization

	Diabetes mellitus (n = 313)			Non diabetes (n = 426)		
	Success (n = 265)	Failed (n = 48)	P value	Success (n = 354)	Failed (n = 72)	P value
Age (years)	70 ± 12	70 ± 13	0.71	66 ± 14	68 ± 13	0.10
Gender (male)	229 (86)	42 (88)	1	339 (96)	65 (90)	0.08
Hypertension	226 (85)	42 (88)	0.83	241 (68)	54 (75)	0.27
Hyperlipidemia	121 (46)	26 (54)	0.35	146 (41)	27 (38)	0.60
Prior stroke	25 (9)	8 (17)	0.13	26 (7)	3 (4)	0.45
PAD	18 (7)	7 (15)	0.08	16 (5)	4 (6)	0.76
Smoking	92 (35)	17 (35)	1.00	161 (46)	30 (42)	0.60
Prior MI	77 (29)	15 (31)	0.73	58 (16)	9 (13)	0.48
Prior PCI	151 (57)	24 (50)	0.43	166 (47)	42 (58)	0.09
Prior CABG	28 (11)	8 (17)	0.22	33 (9)	7 (10)	0.83
LVEF (%)	47 ± 12	47 ± 12	0.92	50 ± 12	48 ± 14	0.36
eGFR, MDRDc (ml/min)	78 ± 36	67 ± 32	0.06	89 ± 29	82 ± 34	0.06
LDL (mg/dl)	98 ± 33	92 ± 30	0.39	106 ± 36	100 ± 34	0.29
HbA1C	7.9 ± 1.7	7.7 ± 1.7	0.57	–	–	–
Non invasive test			0.616			0.068
Treadmill	50 (19)	6 (13)		80 (23)	7 (10)	
Thallium scan	202 (77)	40 (83)		247 (70)	61 (85)	
Others	12 (5)	2 (4)		27 (7)	4 (5)	
OAD	191 (72)	32 (67)	0.62	–	–	–
Metformin	39 (15)	6 (13)				
Sulphonylurea	15 (6)	5 (11)				
DPP4 inhibitor	5 (2)	5 (11)				
Meglitinide	1 (1)	1 (2)				
Combined regimen	81 (31)	12 (25)				
Combined insulin and OAD	31 (12)	4 (8)				
Insulin (%)	55 (21)	13 (27)	0.62	–	–	–
Location of CTO						
LAD	107 (40)	16 (33)	0.42	164 (46)	30 (42)	0.52
LCx	68 (26)	16 (33),	0.29	91 (26)	21 (29)	0.56
RCA	138 (52)	30 (63)	0.21	155 (44)	33 (46)	0.80
J CTO score	2.54 ± 1.00	2.66 ± 1.00	0.38	2.31 ± 0.99	2.40 ± 0.89	0.537
Syntax score	18 ± 6	20 ± 7	0.03	19 ± 6	20 ± 7	0.24
MVD	224 (85)	46 (96)	0.04	277 (78)	60 (83)	0.43
Lesion length (mm)	38 ± 18	31 ± 9	0.12	38 + 18	32 + 12	0.05
Lesion width (mm)	3.0 ± 1.6	3.0 ± 0.6	0.96	3.3 + 4.5	3.0 + 0.3	0.72
Primary retrograde	22 (9)	2 (5)	0.85	26 (8)	6 (8)	1.00

Values are given as mean and standard deviation or numbers and percentages

*PCI* percutaneous coronary intervention, *PAD* peripheral arterial disease, *MI* myocardial infarct, *PCI* percutaneous coronary intervention, *CABG* coronary artery bypass graft surgery, *LVEF* left ventricular ejection fraction, *eGFR* estimated glomerular filtration rate, *MDRDc* modification of diet in renal disease Chinese, *LDL* low density lipoprotein, *HbA1C* glycated hemoglobin, *OAD* oral anti-diabetic drug, *CTO* chronic total occlusion, *CAD* coronary artery disease, *LAD* left anterior descending, *LCx* left circumflex, *RCA* right coronary artery, *MVD* multivessel significant coronary artery disease

**Fig. 3:**
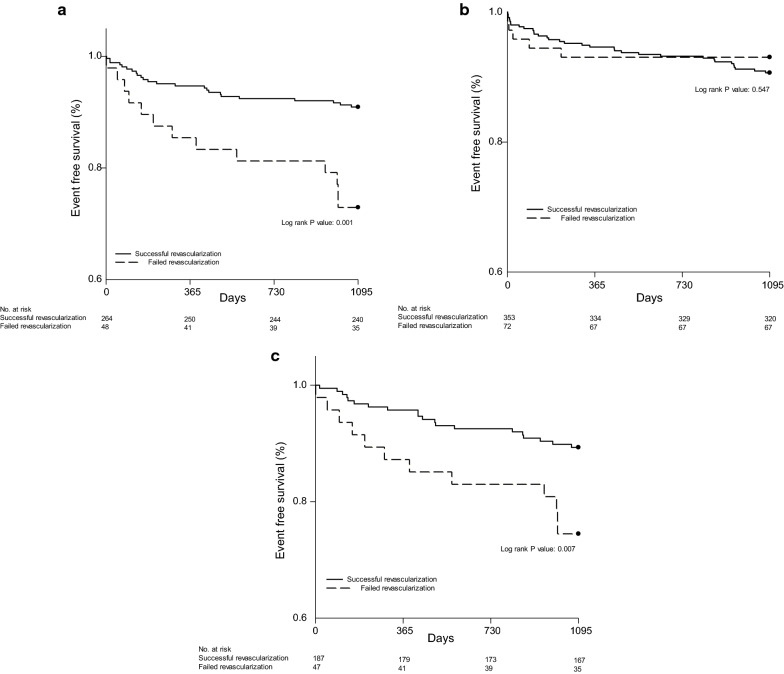
Kaplan Meier survival curves for 3-years all cause mortality of **a** diabetes patients, **b** non diabetes patients, **c** diabetes patients after propensity score matching

Risk of long-term all-cause mortality, CV mortality and MACE in successful recanalization group were significantly lower comparing to those of failed group in diabetics subgroup (HR: 0.307, 95% CI 0.156–0.604, P: 0.001; HR: 0.266, 95% CI 0.095–0.748, P: 0.013; HR: 0.454, 95% CI 0.246–0.837, P: 0.011 respectively), whereas there were no significant differences in these endpoints in non-diabetes population (all-cause mortality: HR: 1.334, 95% CI 0.521–3.417, P: 0.548; CV mortality: HR: 0.885, 95% CI 0.252–3.107, P: 0.849; nonfatal MI: HR: 1.423, 95% CI 0.175–11.565, P: 0.741; and MACE: HR: 1.351, 95% CI 0.573–3.188, P: 0.491) (Table 2). Figure 3 shows the cumulative survival curves free from 3-year all-cause mortality determined using the Kaplan–Meier method between successful and failed revascularization group in entire population and diabetic/non-diabetic patients, with the outcome significantly worse only in those diabetic patients undergoing failed revascularization procedure (P: 0.001). Periprocedural complications were summarized in Table 4. There was no statistically significant difference in complication rate between two groups of diabetes. In non-diabetics, there was higher prevalence of pericardial effusion which required pericardiocentesis in failure group (P: 0.04).

**Table 4:** Periprocedural complications

	Diabetes patients			Non-diabetes patients		
	Successful PCI (n = 265)	Failed PCI (n = 48)	P value	Successful PCI (n = 354)	Failed PCI (n = 72)	P value
In hospital mortality	1 (1)	1 (2)	0.28	3 (1)	2 (3)	0.07
Pericardial effusion required pericardiocentesis	2 (1)	0 (0)	0.72	2 (1)	3 (4)	0.04
Cardiogenic shock required mechanical support	2 (1)	0 (0)	0.72	3 (1)	1 (1)	0.53
CIN required renal replacement therapy	2 (1)	0 (0)	0.72	1 (1)	0 (0)	0.83
Vascular complications required surgery	3 (1)	1 (2)	0.49	3 (1)	0 (0)	0.57
Periprocedural MI	5 (2)	3 (6)	0.11	6 (2)	0 (0)	0.33

*MI* myocardial infarct, *CIN* contrast induced nephropathy

### Propensity score-adjusted clinical outcomes

To reduce the effect of treatment selection bias and compensate for potential confounding factors in this observational study, we calculated the propensity score by using multiple logistic regression analysis incorporating patient’s age, gender, renal function variables. After propensity score matching, there were no significant differences in the baseline characteristics between the successful PCI and failed PCI group of diabetes population (Table 5). In propensity score matched population, successful CTO revascularization was associated with reduced 3-years all-cause mortality and CV mortality only in diabetes population (all-cause mortality; HR 0.386, 95% CI 0.188–0.789, P: 0.009, CV mortality; 0.280, 95% CI 0.094–0.834, P: 0.018, Fig. 3c). In contrast, the risks of non-fatal MI and MACE were not reduced after successful CTO recanalization in diabetic patients (HR: 0.584, 95% CI 0.154–2.210, P: 0.429; HR: 1.511, 95% CI 0.338–6.753, P: 0.589, Table 2). In addition, there were no significant differences in clinical outcomes after successful or failed CTO recanalization in propensity score matched non-diabetic group.

**Table 5:** Baseline demographics and angiographic characteristics of diabetes population with successful and failed PCI after propensity score matching

	Success (n = 188)	Failed (n = 47)	P value
Age, years	71 ± 12	71 ± 13	0.96
Gender (Male)	158 (84)	41 (87)	0.66
Hypertension	160 (85)	42 (89)	0.64
Hyperlipidemia	80 (43)	24 (51)	0.33
Prior stroke	19 (10)	6 (13)	0.60
PAD	16 (9)	7 (15)	0.27
Smoking	61 (32)	17 (36)	0.73
Prior MI	56 (30)	16 (34)	0.60
Prior CAD	111 (59)	24 (51)	0.33
Prior CABG	18 (10)	9 (19)	0.08
LVEF, %	47 ± 12	47 ± 12	0.92
eGFR, MDRDc (ml/min)	69 ± 29	68 ± 30	0.76
LDL (mg/dl)	98 ± 34	93 ± 29	0.51
HbA1C, %	7.7 ± 1.6	8 ± 1.5	0.49
Location of CTO			
LAD	14 (26)	5 (39)	0.50
LCx	28 (44)	1 (11)	0.08
RCA	23 (37)	10 (56)	0.18
Multivessel CTO	29 (15)	15 (32)	0.05
J CTO score	2.4 ± 0.5	2.5 ± 0.6	0.60
Syntax score	18 ± 5	19 ± 5	0.78
MVD	163 (87)	45 (96)	0.05
Lesion length (mm)	37 ± 18	31 ± 8	0.12
Lesion width (mm)	3.0 ± 2.0	3.0 ± 0.6	0.92
Retrograde approach	14 (8)	2 (5)	0.89

Values are given as mean and standard deviation or numbers and percentages

*PCI* percutaneous coronary intervention, *PAD* peripheral arterial disease, *CABG* coronary artery bypass graft surgery, *LVEF* left ventricular ejection fraction, *eGFR* estimated glomerular filtration rate, *LDL* low density lipoprotein, *CTO* chronic total occlusion, *CAD* coronary artery disease, *LAD* left anterior descending, *LCx* left circumflex, *RCA* right coronary artery, *MVD* multivessel significant coronary artery disease

In multivariate Cox-regression analysis, successful CTO revascularization remained an independent predictor of 3 years all-cause mortality in diabetic patients (HR: 0.289, 95% CI 0.125–0.667, P: 0.004) after adjusting age, renal function, prior stroke, prior peripheral arterial disease, left ventricular ejection fraction (Table 6). The results of univariate analysis and multivariate analysis were similar after propensity score matching (Table 7).

**Table 6:** Univariate and multivariate analysis of successful revascularization on 3-years all cause mortality before matching

	Univariate analysis		Multivariate analysis	
	HR (95% CI)	P value	HR (95% CI)	P value
Successful revascularization	0.307 (0.156–0.604)	0.001	0.289 (0.125–0.667)	0.004
Age	1.044 (1.015–1.075)	0.003	1.034 (0.997–1.073)	0.071
Gender	3.607 (0.873–14.902)	0.076	–	–
eGFR	0.990 (0.981–1.000)	0.053	0.996 (0.985–1.007)	0.465
LDL	0.991 (0.979–1.003)	0.150	–	–
HbA1C	0.830 (0.637–1.081)	0.166	–	–
Prior stroke	2.409 (1.158–5.012)	0.019	1.961 (0.725–5.308)	0.185
Prior PAD	3.109 (1.494–6.469)	0.002	3.322 (1.276–8.646)	0.014
Prior MI	1.147 (0.576–2.282)	0.697		
Hypertension	1.302 (0.513–3.302)	0.579	–	–
Multivessel disease	1.205 (0.746–1.946)	0.445		
LAD	1.575 (0.872–2.846)	0.132	–	–
Syntax score	1.054 (0.995–1.117)	0.074		
LVEF	0.960 (0.933–0.988)	0.005	0.955 (0.927–0.984)	0.003

*MDRDc* Modification of diet in renal disease Chinese, *LDL* low density lipoprotein, *HbA1C* glycated hemoglobulin, *PAD* peripheral arterial disease, *LAD* left anterior descending, *LVEF* left ventricular ejection fraction

**Table 7:** Univariate and multivariate analysis of successful revascularization on 3-years all cause mortality after matching

	Univariate analysis		Multivariate analysis	
	HR (95% CI)	P value	HR (95% CI)	P value
Successful revascularization	0.386 (0.188–0.789)	0.009	0.348 (0.142–0.851)	0.021
Age	1.045 (1.010–1.081)	0.012	1.041 (0.997–1.087)	0.069
Gender	2.789 (0.667–11.673)	0.160	–	–
eGFR	0.987 (0.975–0.999)	0.040	0.995 (0.979–1.011)	0.516
LDL	0.993 (0.980–1.007)	0.346	–	–
HbA1C	0.671 (0.386–1.169)	0.159	–	–
Prior stroke	2.598 (1.123–6.009)	0.026	2.207 (0.736–6.621)	0.158
Prior PAD	1.825 (0.703–4.740)	0.217	–	–
Prior MI	1.176 (0.567–2.440)	0.662		
Hypertension	1.603 (0.488–5.261)	0.437	–	–
Multi vessel disease	0.680 (0.226–2.048)	0.493		
LAD	0.194 (0.024–1.577)	0.125	–	–
Syntax score	1.024 (0.888–1.180)	0.745		
LVEF	0.965 (0.933–0.999)	0.045	0.961 (0.928–0.996)	0.027

*MDRDc* Modification of diet in renal disease Chinese, *LDL* low density lipoprotein, *HbA1C* glycated hemoglobulin, *PAD* peripheral arterial disease, *LAD* left anterior descending, *LVEF* left ventricular ejection fraction

## Discussion

Our study showed that though diabetic patients were associated with more co-morbidities and more complex coronary lesions, the CTO revascularization successful rate was similar comparing to that of non-diabetic population. Moreover, successful CTO recanalization was independently associated with reduced risks of all-cause mortality rate and adverse cardiovascular events only in diabetic patients, but not in non-diabetic population. These results remained similar in propensity score matching analysis.

Most studies showed that successful recanalization of CTO reduced long term mortality compared to failed procedure or medical therapy and had comparable clinical results to those receiving bypass surgery [13–15]. But, some studies showed conflicting results [4, 16]. Decision CTO trial reported that there was no difference in long term outcome of successful CTO PCI and optimal medical therapy [4]. However, in this trial, there was high rate of crossover of medical therapy to CTO PCI. Moreover, mean age of patient population was relatively younger (62 years) and proportion of patients with diabetes mellitus (32%) was lower compared to that of previously reported observational studies [14].

Sanguineti et al. found that diabetes mellitus was a significant predictor of cardiac mortality in patients with CTO lesion. CTO recanalisation reduced major adverse cardiovascular event and suggested a greater reduction in cardiac death among diabetic patients [17]. Failure to recanalize CTO lesion in diabetes was found to have higher residual platelet reactivity (HRPR) which may in turn increase cardiac mortality [18]. In the present study, we found similar result that diabetes mellitus was related to poor prognosis in patients with CTO lesions compared to that of non-diabetics. Successful revascularization of CTO lesions in diabetes patients reduced all-cause mortality. But survival benefit was not found in non-diabetics.

Type 2 diabetes mellitus alters glucose and lipid metabolism, leading to premature development of atherosclerosis and adverse outcomes. Prompt recruitment for collateral circulation is crucial to reduce myocardial damage after coronary artery occlusion. Sen et al. found out that diabetes was related to higher incidence of inadequate collateral development in acute coronary syndrome [19]. Potential mechanisms for collateral developments were arteriogenesis, (i.e., arterialization of capillary bed) and angiogenesis [20]. However, chronic hyperglycemia induced microvascular rarefaction in myocardium. It also increases and accumulates advanced glycation endproducts which in turn have negative impact on endothelial function and angiogenesis [21, 22]. Dyslipidemia which is frequently associated with diabetes mellitus confers further greater risk for coronary collateralization [23]. Moreover, diabetes mellitus was associated with diffuse atherosclerosis of donor coronary arteries and further impaired collateral circulation over time [24]. These may be possible reasons behind the benefit after successful revascularization in diabetics.

In our study, diabetes patients were older (mean age was 70 years old) and there was high prevalence of chronic kidney disease and heart failure with reduced ejection fraction. Samy et al. found that left ventricular ejection fraction improvement after successful PCI was significantly more in patient with lower ejection fraction group [25]. Galassi et al. also reported that successful revascularization in patients with left ventricular ejection fraction ≤ 35% improved left ventricular ejection fraction and 2 years all-cause mortality [26]. Moreover, successful PCI was associated with better cardiac survival in elderly especially when complete revascularization is achieved [27]. Recently, Yunfeng et al. also reported that successful revascularization of CTO of stable right coronary artery either by PCI or bypass graft showed significant reduction of all-cause mortality (HR: 0.429, 95% CI 0.269–0.682) [28]. These evidences highlighted the importance of complete revascularization. Benefit of recanalization of CTO may be more pronounced in patients with elderly and poor left ventricular ejection fraction. Chronic kidney disease was one of poor prognostic factors for patients with CTO [29]. Diabetes mellitus is one of well-known underlying diseases that lead to chronic kidney disease. However, successful CTO PCI was associated with better survival irrespective of renal function status of patient [30].

Coronary revascularization of CTO lesion is always complex and demands delicate techniques, ample experiences and familiarity to special devices. Moreover, it is associated with higher perioperative complications such as coronary artery perforation, contrast induced nephropathy, radiation hazard, and mortality. Diabetes mellitus is associated with multi-, small vessel, diffuse atherosclerotic disease and higher rate of periprocedural MI, contrast induced nephropathy which may impact on procedure success rate and complications that consequently affect long term outcomes. However, in Bypass Angioplasty Revascularization Investigation 2 Diabetes trial, CTO didn’t increase periprocedural mortality in diabetes patients treated either by PCI or bypass surgery but it was associated with poor prognosis if left untreated [31, 32]. In our study, technical success rate of revascularization of CTO in diabetics was not different as compare to that of non-diabetics with similar peri-procedural complications. OPEN CTO trial was a prospective multi center registry evaluating about procedural success rate and complications [33]. This trial had also shown successful revascularization rate of 86% and reported no difference in technical outcomes between diabetics and non-diabetics. Taken together, these evidences suggest that CTO in diabetes patients should not preclude the CTO PCI attempt.

## Limitations

Our study had some limitations. First, all patients in our study received coronary revascularization for CTO. There was no control group that received optimal medical therapy or coronary artery bypass graft to compare outcome. Second, it is a retrospective, nonrandomized and observational study. Although we performed propensity score matching to reduce potential bias, the result cannot be comparable to that of randomized trial. Third, our study was conducted in a tertiary medical center that performed high volume of percutaneous CTO revascularization. Our result may not be applicable in low volume and less experienced center. Fourth, due to high proportion (91%) of male gender in our study, it’s application on female gender may be limited. Fifth, as some of our patients were referred to local hospital after intervention, some of follow up information may not be available when our research coordinator couldn’t reach them. Sixth, bare metallic stents were implanted in some patients due to personal economic issue or contraindication to prolonged dual antiplatelet therapy. Next generation drug eluting stent and recent trial about short term dual antiplatelet therapy may solve this problem in future. Seventh, patients with non-diabetics were younger and lesser co-morbidities compared to that of diabetics so that longer follow up period may be necessary to find potential benefit from successful CTO PCI to avoid potential type 2 error. Moreover, data about contrast volume and fluoroscopy time is missing.

## Conclusions

The CTO revascularization successful rate was similar between diabetic and non-diabetic population. Successful CTO recanalization was found to be associated with improved clinical outcomes in diabetic patients. However, the benefit of CTO PCI didn’t outweigh the risk of failed procedure in non-diabetics. Further randomized controlled trial and longer term follow up are necessary to confirm our results.

## Data Availability

The dataset used and analyzed during the current study are available from corresponding author on request.

## Abbreviations

CTO: Chronic total occlusion
PCI: Percutaneous coronary intervention
MACE: Major adverse cardiovascular events
TIMI: Thrombolysis in myocardial infarction
DM: Diabetes mellitus
eGFR: Estimated glomerular filtration rate
MDRDc: Modified diet in renal disease equation for Chinese
CART: Controlled antegrade and retrograde subintimal tracking
CV: Cardiovascular
SD: Standard deviation
LVEF: Left ventricular ejection fraction
